# Rubinstein-Taybi syndrome: a rare case report of a female child emphasizing physiotherapy on gross motor function

**DOI:** 10.11604/pamj.2021.40.85.31240

**Published:** 2021-10-08

**Authors:** Rakesh Krishna Kovela, Mohammad Irshad Qureshi, Ansar Manakandathil, Mukesh Kumar Sinha, Neethu Dinesh, Pallavi Harjpal

**Affiliations:** 1 Department of Neuro-Physiotherapy, Ravi Nair Physiotherapy College, Datta Meghe Institute of Medical Sciences, Sawangi, Meghe, Wardha, Maharashtra, India,; 2 Department of Physiotherapy, AKG Hospital, Perlassery, Kerala, India,; 3 Department of Physiotherapy, Manipal College of Health Professions, Manipal, Academy of Higher Education, Manipal, Karnataka, India,; 4 Clinical Physiotherapist, AKG Hospital, Perlassery, Kerala, India

**Keywords:** Rubinstein-Taybi syndrome, delayed development, physical activities, physiotherapy, case report

## Abstract

Rubinstein-Taybi syndrome (RSTS) is a chromosomal segment 16p13.3 microdeletion syndrome and is characterized by CREBBP gene mutations, delay in the development of height and weight, distinctive facial features, broad and sometimes angulated thumbs and halluces, short stature, and intellectual impairment that is mild to extreme. Current literature emphasizes mainly medical, dental, and psychiatric issues in RSTS and there is no retrievable literature on physiotherapy and its role in improving motor function in RSTS. The present case report is of a baby girl of 17 months suspected case of RSTS, presented with all the features of RSTS. Delay in the acquisition of skills and development were the chief complaints. We designed a 12-week treatment regimen that concentrated mainly on transitions using principles of neurodevelopmental therapy. Gross motor function measure (GMFM 88) was taken pre- and post-treatment which showed tremendous improvement. This is the first study on the role of physiotherapy in RSTS.

## Introduction

Rubinstein-Taybi syndrome (RSTS) is an exceptionally rare genetic disorder first identified in 1963 with autosomal dominance. It has an estimated incidence of one case per 125,000 live births. Typical facial traits, microcephaly, large thumbs and first toes, intellectual disability, and postnatal growth retardation characterize RSTS [1]. The genetic bases were first identified in 1991, showing in some patients a de novo reciprocal translocation with breakpoints in the 16p13.3 chromosome region [2]. Studies have led to the discovery of mutations in the gene encoding the cyclic-AMP-regulated enhancer-binding protein (CREBBP) in 16p13.3 in RSTS patients [3]. About 90 percent of people having disabilities with RSTS live to adulthood, but treatment is especially complicated, time-consuming, and expensive for these patients. Furthermore, for RSTS, no standard diagnostic criteria and follow-up treatment protocols are unavailable. The diagnosis is confirmed by genetic tests and clinical characteristics in most cases [4]. Published data on this condition focus more on clinical features, oral issues [5], dermatology [6], psychiatric issues [7] and other complications because of RSTS. There was no retrievable literature on physiotherapy in RSTS. We believe that there is a significant need to add more information in treatment aspects especially related to RSTS which can help the child to gain motor benefits.

## Patient and observation

**Patient information:** a baby girl of 17 months old who was diagnosed with RSTS by genetic testing presented with parents to the physiotherapy department on recommendation of a pediatrician. She is their first child, and her parents took all the measures to give her the best therapy since the time she got diagnosed with RSTS. She was delivered through the C-section, and everything was normal till the first six months. At that age, the mother noticed some facial changes and took her to the pediatrician, who advised them for genetic testing, which was suspected to be of RSTS. Since then, she was under physiotherapy elsewhere.

**Figure 1:**
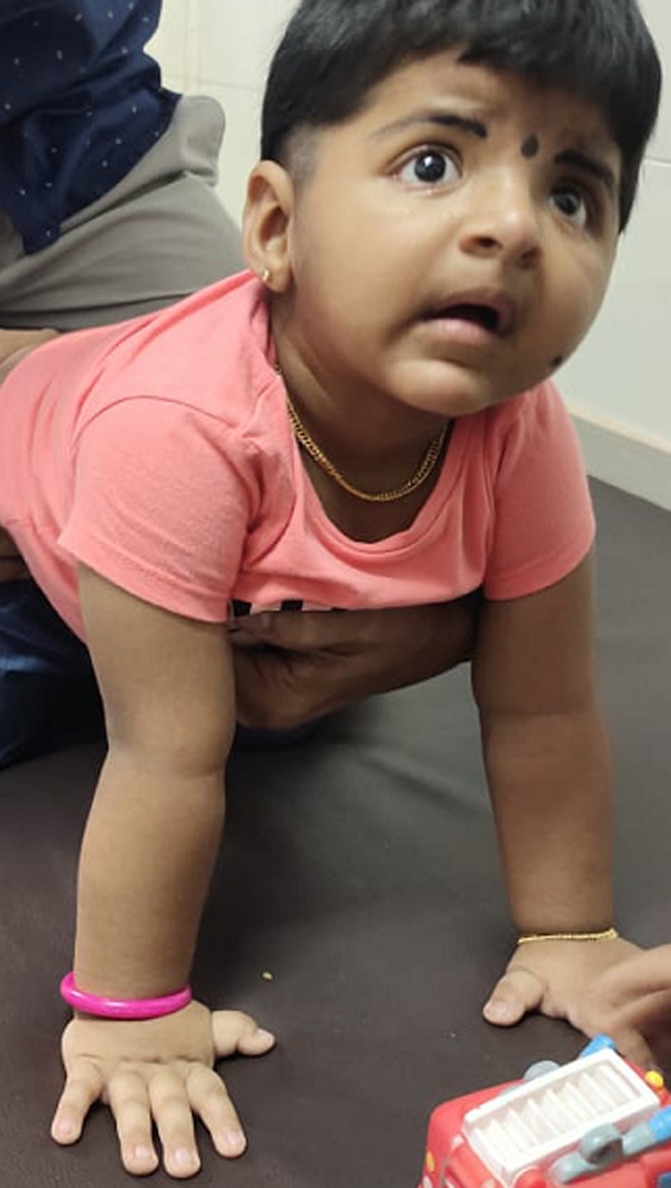
child with features of RSTS

**Clinical findings:** the child presented with dysmorphic facial features, thick eyebrows, micrognathia (Figure 1), and microcephaly. On examination, there were no obvious tonal issues. The gross motor development of the baby was limited to control of the head and neck and partial trunk control. She was able to roll but cannot be able to do it in a controlled manner; she requires assistance to move to a sitting position, there were no issues with vision and auditory sensations.

**Timeline of the current episode:** the complete sequence of events is shown in Table 1, and the child's age-based action plan is shown in the flow map.

**Table 1:** timeline of the events

Gene(Transcript)	Location	Variant	Zygosity	Disease	Inheritance	Classification
CREBBP(+) (ENST00000262367.10)	Exon 30	c.4944dup (p.lle1649HisfsTer11)	Heterozygous	Rubinstein - Taybi syndrome -1	Autosomal dominant	Pathogenic

**Diagnostic assessment:** the genetic testing report is given in Table 1 which explains the genetic transcription, variant, zygosity, disease and inheritance.

**Physiotherapy assessment:** physiotherapeutically, range of motion (ROM) examination was done for bilateral upper and lower extremities which were normal, qualitative assessment of strength for bilateral extremities was poor going to fair, abdominals were weak, developmentally baby was at 5 to 6 months age. Gross motor function measurement GMFM-88 [8] was 18% on the first day of assessment.

**Diagnosis:** Rubinstein-Taybi syndrome (RSTS).

**Therapeutic interventions:** the complete sequence of events is shown in Table 2 and the child's age-based action plan is shown in the flow map. Age-matched goal suggests that she should walk by her present age, which she is unable to do. We have targeted her facilitations by keeping her age in mind. Physiotherapy treatment based on neurodevelopmental treatment (NDT) principles was given one hour a day, five days a week for 12 weeks. As there are no disease-specific outcome measures, we mainly targeted qualitative improvement in various transitions and gait.

**Table 2:** genetic test report depicting disease and inheritance

Consultation	Diagnosis/findings	Treatment/suggestions
Paediatrician	Delayed development	Early intervention
Paediatric Neurologist	Rubinstein Taybi syndrome?	Suggested DNA test, early intervention
Paediatric neurologist	Rubinstein Taybi syndrome?	Suggested immediate DNA test
Paediatric physiotherapist	Rubinstein Taybi syndrome?	Paediatric physiotherapy NDT principles GMFM-88-18%
Paediatric neurologist	Rubinstein Taybi syndrome?	Suggested continuation of physiotherapy
Paediatric physiotherapist	Rubinstein Taybi syndrome?	Increased intensity and frequency of exercises using NDT Principles GMFM-88-38%
Paediatric physiotherapist	Rubinstein Taybi syndrome?	NDT, Gait training GMFM-88-53%
Paediatric physiotherapist	Findings- ease in transitions on bed and out of the bed along with walking	Gait training and home program GMFM -88-61% (goal area)

**Follow-up and outcome of interventions:** the completer sequence of physiotherapeutic interventions and the outcomes are shown in Table 3.

**Table 3:** timeline of physiotherapeutic interventions and outcomes

Goal	Strategy (Using NDT principles)	Duration	Outcome
**Achieve rolling**	Using ATNR (Asymmetric Tonic Neck Reflex), for initiating rolling on an unstable surface (65 cm physio ball) using proximal key points which were progressed to distal key points	3 weeks	By the end of 3 weeks, the child was able to initiate segmental roll by herself
**Transitions from lying to quadruped and sitting**	We Progressed to sitting facilitations (abdominal curls, balance training) on a physio ball (65cm) We used proximal key points over the spine and pelvis to facilitate transitions from lying to quadruped and sitting. We challenged the child by moving from proximal to distal key points to facilitate from lying to quadruped and sitting	3-8 weeks	By the end of 8^th^ week, we found the child is responding well to the treatment i.e., she was able to come to sitting and maintaining static and dynamic balance
**Transitions to standing and walking**	It started with standing training by giving her maximum support at the pelvis and knees and asking the child to stand and perform activities. After two more weeks, we started facilitating her to initiate few steps with minimum support at the pelvis	8-12 weeks	Presently at the end of 12 weeks of treatment, the child is initiating gait with fair to good pelvis and trunk control

**Patient perspective:** the patients´ parents were very cheerful and cooperative during the treatment session. They were pleased with their baby´s recovery and were willing to continue further as the baby enjoyed the sessions.

**Informed consent:** it was obtained from the parents. Parents were happy and overwhelmed with the child´s recovery.

## Discussion

This is the first study on physiotherapy in RSTS. The child's clinical characteristics were compatible with previous research on RSTS. In our case, physiotherapy management was planned based on NDT principles and evaluated through GMFM-88. The child responded to treatment very well. Her compliance to sessions was up to the mark which we feel mainly because of the type of treatment approach and strategies which involved playful activities throughout the sessions. We also attribute our results for the consistency in performing home programs and regular sessions to solve mother´s queries. We observed steady progress week by week. After 12 weeks we were delighted to see the child walking with good trunk and pelvis control with minimal support. Gross motor function measurement 88 score was 61% with tremendous improvement in all five dimensions compared to 18% on day one. Principles of NDT were proved to be very effective in this child which is consistent with previous studies [9]. A previous study on the use of GMFM 88 in cases other than cerebral palsy also reported similar findings [10]. We will be publishing long-term follow-up and the importance of physiotherapy in gaining gross and fine motor activities in near future.

## Conclusion

We would like to conclude by saying that early physiotherapy with a principles-oriented therapeutic regimen like NDT helps in improving gross motor developmental milestones in children with RSTS.
